# The association of Val109Asp polymorphic marker of intelectin 1 gene with abdominal obesity in Kyrgyz population

**DOI:** 10.1186/s12902-018-0242-6

**Published:** 2018-02-27

**Authors:** Jainagul Isakova, Elnura Talaibekova, Denis Vinnikov, Nazira Aldasheva, Erkin Mirrakhimov, Almaz Aldashev

**Affiliations:** 1 Institute of Molecular Biology and Medicine, 3 Togolok Moldo Street, 720040 Bishkek, Kyrgyzstan; 2 School of Public Health, al-Farabi Kazakh National University, 71 Al-Farabi avenue, Almaty, Kazakhstan 050040; 3 I.K. Akhunbaev Kyrgyz State Medical Academy, 92 Akhunbaev Street, Bishkek, 720020 Kyrgyz Republic

**Keywords:** Intelectin 1 gene, Val109Asp polymorphism, Abdominal obesity, Kyrgyz population

## Abstract

**Background:**

The aim of this study was to quantify the association of Val109Asp polymorphism of intelectin 1 (*ITLN1*) gene with the abdominal obesity (AO) in Kyrgyz population.

**Methods:**

Patients admitted to annual screening at a local outpatient facility were enrolled or this study. We genotyped 297 nonrelated adults of Kyrgyz ethnicity, of whom 127 were AO patients, including 46 men and 81 women with the mean age 53.2 ± 7.1 years, and 170 non-obese controls, including 61 men and 109 women with the mean age 52.0 ± 9.0 years. AO was defined as having waist circumferences ≥ 102 cm in men and ≥ 88 cm in women. We used PCR-RFLP method to define Val109Asp polymorphism of *ITLN1* gene.

**Results:**

Asp109Asp, Asp109Val and Val109Val genotypes were found in 48%, 40%, and 12% of AO patients respectively, and in 53%, 43%, and 4% of controls, whereas Val109Val homozygous genotype of *ITLN1* gene Val109Asp polymorphic marker was significantly more prevalent in AO patients. In Kyrgyz population, Val109Val genotype of *ITLN1* gene increased the risk of AO (odds ratio (OR) 3.12, 95% CI 1.23–7.90). Asp109Asp homozygous genotype, on opposite, was not associated with this condition (OR 0.82, 95% CI 0.53–1.30). Finally, the allelic variants of Val109Asp polymorphism of *ITLN1* gene were not associated with AO.

**Conclusion:**

Significant increase in the frequency of Val109Val genotype of *ITLN1* gene in AO patients may be indicative of some potential role of *ITLN1* gene in molding genetic predisposition to AO in the Kyrgyz. This requires further elaboration in the future studies.

## Background

There is a trend towards growing prevalence of overweight and obese people in almost all populations in the twenty-first century [1]. Compared to 80–90s of the last century, the number of overweight people and those with obesity, including abdominal obesity rose almost two-fold, reaching the levels of 30.8%, 25.7% and 52.3% in Kyrgyz Republic [2]. In the last decades, eating patterns and the lifestyle of Kyrgyz has changed with less physical activity and increase in the overall load with fast carbohydrates and saturated fat [3]. Being nomads for centuries with traditional lifestyle and eating habits, Kyrgyz population nowadays experiences the impact of globalized economy and greater role of highly processed foods.

Long-lasting misbalance between calorie intake and energy expenditure will result in the accumulation of visceral adipose tissue, resulting in excess bodyweight and even obesity [4]. Adipose tissue is known to synthesize series of adipocytokines, including leptin, adiponectin, resistin, visfatin, chemerin, vaspin, and apelin which all take part in the carbohydrates and lipids metabolism [5]. Selected adipocytokines synthesized in the adipose tissue may predict quite a range of diseases. Omentin (Intelectin-1: *ITLN1*, OMIM: 609873) is one of recently discovered secretory proteins of visceral adipose tissue with two isoforms identified as omentin-1 and omentin-2 [6]. Omentin may have a serum concentration of 100 to 1 μg/ml [7], dropping to lower levels in patients with obesity, insulin resistance and diabetes type II [7–10]. In these conditions, reduced omentin levels correlate with increased body mass index (BMI), insulin resistance **(**НОМА-IR), high triglycerides (TG) level and low-density lipoprotein cholesterol [7, 8]. Low blood omentin concentration accompanied reduction in adiponectin and high-density lipoprotein cholesterol levels [9, 10].

Omentin is a 313-amino acid protein with a molecular mass of 38 Kd [11]. The gene to code its amino acid sequence is located in the 1q22-q23 locus of the first chromosome and comprises 8 exons and seven introns [12]. There exists a polymorphic locus in the position 326 of the fourth exon, where adenine is replaced with thymine (326 GAC → GTC, rs2274907), which entails the replacement of asparagine with valine in the position 109 of *ITLN1* (Val109Asp) [12].

At present, the evidence on the association of *ITLN1* gene with obesity is very poor, with only very few studies published [12–14]. To our best knowledge, there are no existing publications on the prevalence of genotypes and alleles of *ITLN1* gene Val109Asp polymorphic marker in Kyrgyz population, as well as on its association with abdominal obesity (AO) in Kyrgyz adults, whereas AO in its turn is closely linked with cardiovascular complications. The aim of this study was to ascertain the risk of AO in Kyrgyz population associated with *ITLN1* gene Val109Asp polymorphism.

## Methods

### Participants, recruitment and study design

In this case-control study, we enrolled 297 unrelated subjects with known Kyrgyz ethnic background, of whom 127 subjects were cases, including 46 men and 81 women with the mean age 53.2 ± 7.1 years. Subjects were enrolled from the annual screening at a local outpatient facility in Bishkek, where they routinely applied for fitness certificates. Cases had AO, when both body mass index (BMI) exceeded 30 kg/m^2^ and waist circumference exceeded 88 cm in women and 102 cm in men (FDA criteria, 2005). One hundred and seventy healthy subjects, of whom 61 were men and 109 women with the mean age 52.0 ± 9.0 with no known history of AO, cardiovascular diseases and with normal carbohydrate and lipid metabolism were controls.

Both cases and controls in this study were subjected to a uniformed clinical examination, which included anamnesis, anthropometric tests (height, body mass, waist circumference (WC), hip circumference (HC)) with the subsequent calculation of BMI, and blood pressure (BP) measurement (systolic (SBP) and diastolic (DBP). BMI was calculated as height (m)/body mass (kg)^2^. Obesity was diagnosed with BMI ≥ 30 kg/m^2^. Subjects with WC ≥ 88 cm in women and ≥102 cm in men were diagnosed with AO.

We used standard techniques to measure blood sugar, serum total cholesterol (TC), triglycerides (TG), and high-density lipoprotein cholesterol (HDLC) using “Beckman” biochemical analyzer (USA). We used Friedwald formula to calculate low-density lipoprotein cholesterol (LDLC), whereas serum immunoreactive insulin was tested with immunuenzyme analysis in the Hospital Saint-Vincent De Paul’s lab (Paris, France). Homeostasis Model Assessment of Insulin Resistance (HOMA-IR) was calculated using the formula: HOMA-IR = serum insulin (μIU/ml) * serum glucose (mmol/l)/22.5. We considered IR present when HOMA-IR was equal to or exceeded 2.77.

### DNA extraction and genotyping

**Fig. 1:**
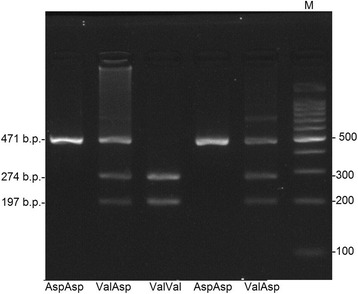
Electrophoretic separation of Val109Asp polymorphic locus genotypes of *ITLN1* gene in 3*%* agarose gel. Homozygous genotype of minor Val/Val allele is DNA fragment with the length of 274 and 197 bp; Val/Asp heterozygous genotype 471, 274 and 197 bp long; homozygous genotype of Asp/Asp widespread allele 471 bp long; М is a DNA marker of molecular scales with 100 bp shift

DNA was extracted from peripheral blood white cells via a standard two-stage phenol-chlorophorm method. We then used 5’**-**GAGCCTTTAGGCCATGTCTCT**-**3’ and 5’**-**CTCTCCTTCTTCTCCAGCCCAT**-**3’ primers to amplify Val109Asp polymorphic locus of the *ITLN1* gene. The *ITLN1* gene amplified locus with a length of 471 base pairs (bp) was split into fragments using AccI endonuclease. PCR and restriction data were detected with 3*%* agarose gel and then photographed by means of UVP (UK) gel documenting system (Fig. 1).

### Statistical analysis

We used STATISTICA v.7.0. (StatSoft) and GraphPad Prism v 5.0 to run all the statistical tests for this report. We report quantitative data as mean ± standard deviation, or as median with the corresponding interquartile range, whereas qualitative data are summarized as absolute number of patients with the corresponding percent of those in the group. In both cases and controls, we tested the frequencies of alleles, allele carriage, as well as genotype frequencies. The deviations of genotype frequencies from Hardy-Weinberg’s equilibrium were verified with a Haploview 4.0 free software [http://www.broad.mit.edu/mpg/haploview]. The frequencies of alleles, allele carriage, as well as genotype frequencies in cases were compared to controls with Fischer exact test in the online version of GraphPad Instat program [http://www.graphpad.com/quickcalcs/index.cfm]. Data are presented as absolute numbers of patients with a given genotype and its corresponding percent in the group. Associations were quantified with the odds ratio (OR) and their corresponding 95% confidence intervals (CI). The probability of chance lower than 5% (*p* < 0.05) was considered significant.

## Results

The clinical and biochemical characteristics of patients are shown in Table 1. There were no statistically significant differences between the groups in terms of sex and age. Patients with AO showed significant lipid and carbohydrate metabolism deviations such as fasting blood glucose, TG and LDLC levels increase, with the corresponding reduction in HDLC. Forty-six (36%) out of 127 patients with AO had IR, 95 (75%) had obesity, 69 (54%) had type 2 diabetes, 69 (54%) had essential hypertension and 114 (90%) had metabolic syndrome (MS).

**Table 1:** Baseline clinical and biochemical data of cases and controls

Index	Controls, *n* = 170	Cases, *n* = 127	*p*
Age, years	52.0 ± 9.0	53.2 ± 7.1	NS
Sex (male), n (%)	61 (36%)	46 (36%)	NS
Weight, kg	64.7 ± 9.8	83.5 ± 14.1	0.00001
Essential hypertension, n (%)	0	69 (54.3%)	–
SBP, mm Hg	122.0 ± 10.6	148.6 ± 27.0	0.00001
DBP, mm Hg	79.3 ± 9.3	91.7 ± 14.5	0.00001
Type 2 diabetes, n (%)	0	69 (54%)	–
Blood glucose, mmol/l	5.20 [5.01;5.48]	7.33 [5.49;8.48]	0.001
MS, n (%)	0	114 (90%)	–
Obesity, n (%)	0	95 (75%)	–
BMI, kg/m^2^	24.1 ± 2.7	31.5 ± 4.1	0.00001
WC (cm)	83.2 ± 8.6	104.1 ± 9.2	0.00001
HC (cm)	95.6 ± 7.8	109.5 ± 8.6	0.00001
TC, mmol/l	5.0 ± 0.9	5.1 ± 1.0	NS
HDLC, mmol/l	3.2 ± 0.8	1.0 ± 0.3	0.00001
LDLC, mmol/l	1.3 ± 0.4	3.2 ± 0.9	0.00001
TG, mmol/l	1.2 ± 0.7	2.1 ± 1.5	0.00001
НОМА-IR	0	46 (36%)	–
Insulin, μIU/ml	5.08 [3.35;6.65]	12.7 [7.52;15.90]	0.001

*MS* metabolic syndrome, *WC* waist circumference, *HC* hip circumference, *SBP* systolic blood pressure, *DBP* diastolic blood pressure, *BMI* body mass index, *TC* total cholesterol, *HDLC* high-density lipoprotein cholesterol, *LDLC* low-density lipoprotein cholesterol, *TG* triglycerides, *NS* non-significant, *р* probability value

The frequency distribution of Val109Asp polymorphism of *ITLN1* gene in controls (χ^2^ = 0.725; *p* = 0.394) and in cases (χ^2^ = 2.768; *p* = 0.09) corresponded to Hardy-Weinberg’s equilibrium. When testing Val109Asp locus of *ITLN1* gene in cases in controls, we identified three possible combinations: Val/Val = GTC/GTC, Val/Asp = GAC/GTC and Asp/Asp = GAC/GAC (Table 2). About 50% of subjects in Kyrgyz population, therefore, carry homozygous genotype of the widespread (major) Asp109Asp allele.

**Table 2:** The genotype and allele frequencies of Val109Asp polymorphic marker of *ITLN1* gene in cases compared to controls

Genotypes and alleles	Cases, *n* = 127 (%)	Controls, *n* = 170 (%)	χ^2^; p	OR (95% CI)
Asp109Asp	61 (48)	90 (53)	χ^2^ = 6.29; *p* = 0.043	0.82 (0.53–1.30)
Val109Asp	51 (40)	73 (43)		0.89 (0.56–1.42)
Val109Val	15 (12)	7 (4)		3.12 (1.23–7.90)
Asp	173 (68)	253 (74)	χ^2^ = 2.54; *p* = 0.11	0.73 (0.51–1.05)
Val	81 (32)	87 (26)		1.36 (0.95–1.95)

*OR* odds ratio, *CI* confidence interval; data are presented as absolute number of patients with their percent in the groups, whereas ORs presented with their corresponding 95% CI. For ORs, the reference group is a pool of two alternative genotypes

In the univariate comparative analysis, we found statistically significant greater prevalence of homozygous genotype of the less spread (minor) Val109Val allele. The frequency of minor and major alleles in controls was 0.26 and 0.74, and 0.32 и 0.68 in patients with AO (χ^2^ = 2.54, *p* = 0.11).

## Discussion

This case-control study confirmed the association of Val109Asp polymorphic marker of *ITLN1* gene with AO in Kyrgyz population. Whenever homozygous Val109Val genotype is present, the likelihood of AD increases 3-fold (OR = 3.12; 95% CI 1.23–7.90). In the given population, significant increase in the frequency of Val109Val genotype of *ITLN1* gene in AO patients may be indicative of the association of *ITLN1* gene with AO, which requires further investigation.

Statistical power of our case-control study is expected to reach 100%, based on a conventional power calculation for this type of studies. With a number of cases *N* = 127, an approximate case/controls ratio 1:1, Z-alpha 1.96 and the OR of the association extracted from the preceding study (OR = 4.5), as well as the percent of exposed controls 10%, the number of patients in our study dramatically exceeds the patients number needed to achieve 80% power (*N* = 38).

In general, genetic predisposition is a well-studied risk factor for obesity [15], which in turn, especially its abdominal form, is a major risk factor for type 2 diabetes, essential hypertension and MS [1, 16, 17]. At present, adipocytokine family genes attract much attention to study such genetic predisposition to AO and related conditions, whereas their products, secreted with the visceral adipose tissue and being the link to various metabolic and cellular processes, may directly or indirectly affect carbohydrate and lipid metabolism [6, 15]. *ITLN1* gene is one of those recently identified genes, whose product is selectively secreted by the visceral adipose tissue. In 2005, Schaffler et al. [11] studied and published the genome structure, nucleotide sequence, promoter, and exon-intron structure of *ITLN1* gene. There were two mononucleotide polymorphisms identified in the fourth exon of *ITLN1* gene, including A326T (Val109Asp, rs2274907) and С258Т (His86His, rs2274908) [12].

At present, there are only a few studies of the distribution of Val109Asp polymorphism of *ITLN1* gene in the European and Asian populations. Thus, minor allele of Val109Asp marker of *ITLN1* gene had a 0.18 prevalence in Iranian population [13], 0.22 in Turkish population [14], 0.30 in Czech [18], and 0.31 in subjects with German origin [12]. Our data showed that the prevalence of minor allele in Kyrgyz population does not significantly differ from other populations and equals 0.26. Data from this study in comparison with other studies in terms of minor allele frequency (MAF) and the corresponding data are shown in Table 3. When comparing data from other populations, we conclude that, in general, individuals with major allele prevail in total population (72–88% subjects), whereas 18–31% carry minor allele.

**Table 3:** Allele frequency and genotype distribution Val109Asp polymorphic marker of *ITLN1* gene in different populations

Populations	HWE, *P* value	Allele	Allele frequency	Genotype	Genotype frequency (%)	Author(s)
Kyrgyz (*n* = 170)	*p* = 0.09	Asp	0.74	Asp/Asp	90 (53.0)	This study
		Val	0.26	Val/Asp	73 (43.0)	
				Val/Val	7 (4.0)	
Czech (*n* = 495)	*p* = 0.72	Asp	0.70	Asp/Asp	240 (48.0)	Splichal et al., 2015 [18]
		Val	0.30	Val/Asp	207 (42.4)	
				Val/Val	48 (9.6)	
Turkey (*n* = 42)	p = 0.04	Asp	0.76	Asp/Asp	22 (52.3)	Yaykasli et al., 2013 [22]
		Val	0.24	Val/Asp	20 (47.6)	
				Val/Val	0	
Turkey (*n* = 39)	*p* = 0.28	Asp	0.88	Asp/Asp	25 (64.0)	Turan, et al., 2013 [14]
		Val	0.22	Val/Asp	11 (28.0)	
				Val/Val	3 (8.0)	
Germany (*n* = 276)	*p* = 0.036	Asp	0.72	Asp/Asp	138 (50.0)	Schäffler et al., 2007 [12]
		Val	0.28	Val/Asp	124 (44.9)	
				Val/Val	14 (5.1)	
Iran (*n* = 150)	*p* = 0.60	Asp	0.82	Asp/Asp	101 (67.3)	Kohan, 2014 [13]
		Val	0.18	Val/Asp	43 (28.7)	
				Val/Val	6 (4.0)	

*HWE* Hardy – Weinberg equilibrium

Available literature data on the association of Val109Asp polymorphic locus of *ITLN1* gene with various constituents of MS, including AO, is very scarce. In 2015, a group of authors identified association of Val109Asp locus of *ITLN1* gene with daily energy intake in a sample of subjects with Czech origin [18]. In this study, they found a significant difference in genotype distribution when comparing obese and morbidly obese subjects, where AA genotype was significantly less frequent in the morbidly obese cohort and AT genotype, on opposite, was more frequent in the morbidly obese subjects compared to obese group. In another study, those having Val109Val genotype had almost 5-fold increased probability of obesity compared to Asp109Asp carriers (OR = 4.5; 95% CI 1.3–14.9; *p* = 0.01) [13], whereas minor allele was associated with a 1.5-fold increase in breast cancer risk (OR = 1.5; 95% CI 1–2.20; *p* = 0.04) [19].

Data on the association of polymorphic variants of Val109Asp *ITLN1* gene with the omentin production, functional activity and its blood level are also insufficient. In Czech population, the least omentin blood concentration (409.7 ± 169.1 μg/l) was detected in subjects with Val109Val genotype, as opposed to its highest concentration of 475.7 ± 153.7 μg/l in those carrying Asp109Asp variant. Those findings were not, however, significant [18].

Numerous clinical and experimental studies confirmed that omentin was a multifunctional protein, resembling adiponectin in its biological properties, such as vasodilating, anti-inflammatory, immunocompromising and anti-diabetic effects [20, 21]. Blood omentin levels were significantly lessened in patients with obesity, both types of diabetes, endothelial dysfunction and a few cardiovascular diseases [7, 8, 20]. Low blood omentin levels correlate with high body mass index, WC/HC ratio, HOMA–IR, high blood insulin, glucose, TG and LDLC levels, as well as low adiponectin and HDLC levels [7, 8, 10, 20]. These clinical and experimental findings altogether prompt some omentin’s contribution to MS and series of cardiovascular conditions associated with that. Given that, low blood omentin level is proposed as a valuable predictor of MS and cardiovascular complications [5, 9, 20, 21].

Despite showing significant associations, reaching high statistical power for such study design and being the first study to verify the magnitude of such association in Kyrgyz population, the limitations of this analysis should also be noted. Thus, we could not support our results with the phenotypic analysis of our population. Because we could not perform serum omentin tests, and adjust them for sex, age and other covariates, our findings have a limited clinical relevance and should be treated appropriate to a specific clinical situation. Besides, AO diagnosis was made without body composition measurements, as they were not available, and we consider this another limitation of our analysis.

We need to state that currently full-genome studies in Kyrgyz Republic are not feasible. Studies on multifactorial diseases in our country are still limited with single nucleotide polymorphism search only. The current study is the first and only to assess Val109Asp polymorphism of *ITLN1* gene in the whole Central Asian population, and also one of very few uncovering the association of *ITLN1* gene with AO. This study enabled to test the distribution of genotypes and alleles of *ITLN1* gene’s Val109Asp polymorphism in Kyrgyz population, as well as to detect significant increase in Val109Val genotype frequency in patients with AO. This may support the hypothesis of the role of *ITLN1* in genetic predisposition to AO in the Kyrgyz. Such conclusion clearly needs further confirmation in larger samples and with other genetic markers.

## Conclusions

Significant increase in the frequency of Val109Val genotype of *ITLN1* gene in AO patients may be indicative of some potential role of *ITLN1* gene in molding genetic predisposition to AO in the Kyrgyz. Identification of candidate genes, which are associated with abdominal obesity in various ethnicities, is relevant for the healthcare. Finding such genotypes and alleles, associated with the disease, will help individualize primary and secondary prevention programs for such patients in order to reduce the risk.

## Abbreviations

AO: Abdominal obesity
BMI: Body mass index
BP: Blood pressure
DBP: Diastolic blood pressure
HC: Hip circumference
*ITLN1*: Intelectin 1
MS: Metabolic syndrome
PCR: Polymerase chain reaction
SBP: Systolic blood pressure
WC: Waist circumference
